# Integrating Network Pharmacology with Molecular Docking to Unravel the Active Compounds and Potential Mechanism of Simiao Pill Treating Rheumatoid Arthritis

**DOI:** 10.1155/2020/5786053

**Published:** 2020-11-03

**Authors:** Mengshi Tang, Xi Xie, Pengji Yi, Jin Kang, Jiafen Liao, Wenqun Li, Fen Li

**Affiliations:** ^1^Department of Rheumatology and Immunology, The Second Xiangya Hospital, Central South University, Changsha, Hunan 410011, China; ^2^Department of Integrated Traditional Chinese & Western Medicine, The Second Xiangya Hospital, Central South University, Changsha, Hunan 410011, China; ^3^Department of Pharmacy, The Second Xiangya Hospital, Central South University, Changsha, Hunan 410011, China; ^4^Institute of Clinical Pharmacy, Central South University, Changsha, Hunan 410011, China

## Abstract

**Objective:**

To explore the main components and unravel the potential mechanism of simiao pill (SM) on rheumatoid arthritis (RA) based on network pharmacological analysis and molecular docking.

**Methods:**

Related compounds were obtained from TCMSP and BATMAN-TCM database. Oral bioavailability and drug-likeness were then screened by using absorption, distribution, metabolism, and excretion (ADME) criteria. Additionally, target genes related to RA were acquired from GeneCards and OMIM database. Correlations about SM-RA, compounds-targets, and pathways-targets-compounds were visualized through Cytoscape 3.7.1. The protein-protein interaction (PPI) network was constructed by STRING. Gene Ontology (GO) analysis and Kyoto Encyclopedia of Genes and Genomes (KEGG) pathway enrichment analysis were performed via *R* packages. Molecular docking analysis was constructed by the Molecular Operating Environment (MOE).

**Results:**

A total of 72 potential compounds and 77 associated targets of SM were identified. The compounds-targets network analysis indicated that the 6 compounds, including quercetin, kaempferol, baicalein, wogonin, beta-sitosterol, and eugenol, were linked to ≥10 target genes, and the 10 target genes (PTGS1, ESR1, AR, PGR, CHRM3, PPARG, CHRM2, BCL2, CASP3, and RELA) were core target genes in the network. Enrichment analysis indicated that PI3K-Akt, TNF, and IL-17 signaling pathway may be a critical signaling pathway in the network pharmacology. Molecular docking showed that quercetin, kaempferol, baicalein, and wogonin have good binding activity with IL6, VEGFA, EGFR, and NFKBIA targets.

**Conclusion:**

The integrative investigation based on bioinformatics/network topology strategy may elaborate on the multicomponent synergy mechanisms of SM against RA and provide the way out to develop new combination medicines for RA.

## 1. Introduction

Rheumatoid arthritis (RA) is a chronic polyarticular symmetric disease. It is characterized by chronic inflammation of the synovial membrane, which can destroy articular cartilage and juxta-articular bone [1]. RA affects 0.3%–1% of the population worldwide [2]. If insufficiently treated, it usually leads to persistent joint inflammation, progressive joint destruction, continuing functional decline, extra-articular manifestations, disability, and increased mortality [3, 4]. Although current available therapeutic approaches against RA, including nonsteroidal anti-inflammatory drugs (NSAIDs), disease-modifying antirheumatic drugs (DMARDs), and corticosteroid, allow for excellent disease control, novel therapies are needed because RA remains incurable [5]. Furthermore, the long-term use of these drugs may cause multiple side effects and lead to limited therapeutic responses. Therefore, novel treatments are in urgent demand.

Traditional Chinese medicine (TCM) has been extensively applied for the treatment of RA for centuries in Asia and has been gradually accepted for worldwide clinical applications [6, 7]. Numerous studies have indicated that TCM can be served as complementary and alternative RA drugs for therapeutic effects and with fewer side effects [8, 9].

Simiao pill (SM), a traditional TCM formula, comprises four herbs, including Phellodendri Chinensis Cortex (Huang Bo), *Atractylodes lancea* (Thunb.) Dc. (Cang Zhu), achyranthis bidentatae radix (Niu Xi), and Coicis Semen (Yi Yi Ren). Previous studies have indicated the anti-inflammation pharmacological effect of SM [10] and that SM reduced proinflammatory cytokine production by suppressing nuclear factor kappaB (NF-*κ*B)/pyrin domain containing 3 (NLRP3) inflammasome activation [11]. Recently, SM was demonstrated to exhibit anti-inflammatory and bone-protective effects by regulating autotaxin (ATX)-lysophosphatidic acid (LPA) and mitogen-activated protein kinase (MAPK) signaling pathways in collagen-induced arthritis (CIA) rats [12]. In addition, SM was recommended for the treatment of active RA (53.6%) in the expert consensus regarding the treatment of RA with various Chinese patent medicines (CPMs) [13]. However, because TCM formulas are characterized by multicomponents, multitargets, and multipathways [14], the therapeutic effect of SM against RA has not been fully elucidated. Therefore, it is necessary for further systematic investigation.

Nowadays, network pharmacology integrates network biology and polypharmacology based on existing databases, providing a novel approach for exploring the mechanisms and synergistic effect of TCM formulas as disease treatments [14–16]. Combining network science with ancient TCM formulas to investigate multiple molecular mechanisms has achieved successful attempts in the previous researches [17–20].

Therefore, in this study, a network pharmacology-based study was conducted to predict bioactive compounds and elucidate the comprehensive pharmacological mechanisms about the antirheumatic effect of SM. In addition, molecular docking analysis was performed to validate in silico to predict molecular interactions between compounds and targets.

## 2. Materials and Methods

**Figure 1:**
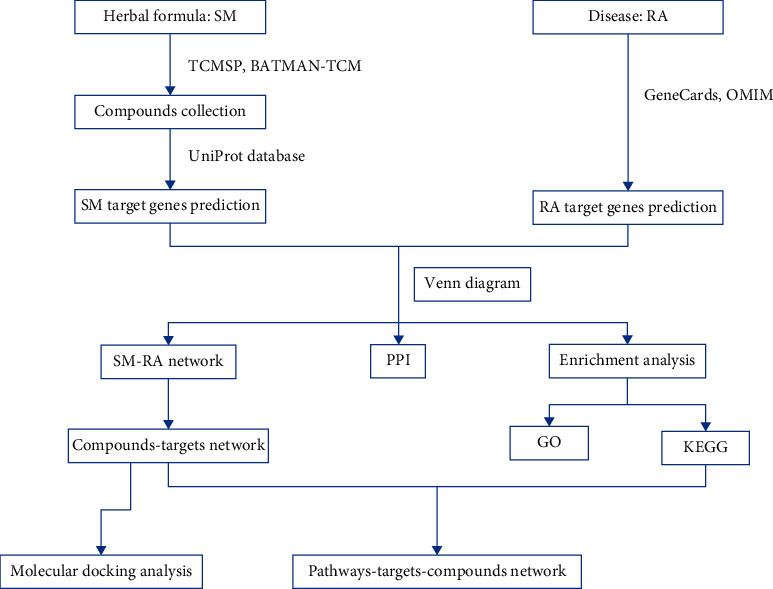
Workflow of network pharmacology analysis.

Network pharmacology-based prediction of SM treating RA was constructed by the following (Figure 1): (1) data collection and preparation, including retrieving the ingredients list of SM formula, screening for candidate compounds, identifying SM and RA targets, and intersecting the identified targets of compounds and disease; (2) topological analysis of network and protein-protein interaction (PPI) network construction; (3) enrichment analysis; and (4) molecular docking analysis.

### 2.1. Data Collection and Preparation

#### 2.1.1. Composite Compounds of SM

The related composite compounds of SM were obtained from the Traditional Chinese Medicine Systems Pharmacology Database (TCMSP, http://lsp.nwu.edu.cn/tcmsp.php) and a Bioinformatics Analysis Tool for Molecular mechANism of Traditional Chinese Medicine (BATMAN-TCM, http://bionet.ncpsb.org/batman-tcm/).

#### 2.1.2. Pharmacokinetic ADME Evaluation

The in silico integrative ADME (absorption, distribution, metabolism, and excretion) model administrated by TCMSP is employed for pharmaceutical research. As an oral drug, two related-ADME models, oral bioavailability (OB), and drug-likeness (DL) are applied to identify the potential bioactive compounds in this study. Only the compounds with OB ≥ 30 and DL ≥ 0.18 that satisfied the criteria suggested by the TCMSP database (removed the duplicated) are retained as the candidate compounds for further study [21]. In addition, among the compounds with OB ＜30 or DL ＜0.18, which are searched with “compound (name)” and “rheumatoid arthritis” [all fields] in PubMed databases to find relevant researches, the compounds in purified form focused on anti-RA mechanisms are also considered to be bioactive compounds (removed the duplicated) and included for further study.

#### 2.1.3. Predictions of Target Genes Related to the Identified Compounds

All the potential compounds were input into TCMSP to capture the relationships between drugs and targets. Since the obtained targets include various biological species, all target names were also put into UniProt databases (http://www.uniprot.org/) to search for target gene names selected by human species.

#### 2.1.4. Potential Disease Target Genes

Information of known RA-related therapeutic target genes was collected by keywords “rheumatoid arthritis” as queries from The Human Gene Databases (GeneCards, https://www.genecards.org/, ver.4.9.0) and Online Mendelian Inheritance in Man (OMIM, http://www.omim.org/, updated June 6, 2019), and only “*Homo sapiens*” target genes linked to RA are selected.

#### 2.1.5. Venn Analysis

All target genes of identified compounds and RA are put into Bioinformatics and Evolutionary Genomics system (bioinformatics.psb.ugent.be/webtools/Venn/), respectively, to produce a Venn diagram, which indicates the intersection of identified targets of drug and disease.

### 2.2. Topological Analysis of Network and PPI Network Construction

#### 2.2.1. Topological Network Analysis

SM-RA mechanism network, compounds-targets network, and pathways-targets-compounds network were visualized through Cytoscape (https://cytoscape.org/, ver. 3.7.1) to systemically explore the molecular mechanisms of SM treating RA.

#### 2.2.2. PPI Network Construction

The above 77 target genes acquired from the Venn diagram intersection were imported into STRING (https://string-db.org/, version 11.0) to construct a PPI network for understanding protein interaction systematically. The PPI network is constructed by setting the organism as “human sapiens”, setting the minimum required interaction score to “medium confidence (0.40)”, and excluding the disconnected protein nodes. In addition, statistics of protein interactions are figured out according to the PPI network, and a related bar plot diagram is constructed with *R* 3.6.0 subsequently.

### 2.3. Enrichment Analysis

R 3.6.0 and related *R* packages (colorspace, stringi, DOSE, clusterProfiler, and pathview) are applied to carry out Gene Ontology (GO) enrichment analysis and Kyoto Encyclopedia of Genes and Genomes (KEGG) pathway enrichment analysis of intersection target genes of SM-RA. *P* values ＜0.05 and q values ＜0.05 are considered statistically significant based on Fisher's test.

### 2.4. Molecular Docking Analysis

The 3D structures of candidate targets were obtained from the PDB database (http://www.rcsb.org/) in PDB format by setting the organism to “*Homo sapiens* only”. The 3D conformers of candidate compounds are acquired from the PubChem database (https://pubchem.ncbi.nlm.nih.gov/) with SDF format. Subsequently, they were imported to the Molecular Operating Environment (MOE) to get the docking score. The greater the absolute value of the docking score, the better.

## 3. Results

### 3.1. Data Collection and Preparation

#### 3.1.1. Identification of Compounds in SM

A total of 479 compounds were identified in SM, including 140 in Phellodendri Chinensis Cortex (Huang Bo), 49 in *Atractylodes lancea* (Thunb.) Dc. (Cang Zhu), 176 in achyranthis bidentatae radix (Niu Xi), and 38 in Coicis Semen (Yi Yi Ren), and in TCMSP, also including 37 in Phellodendri Chinensis Cortex (Huang Bo), 26 in *Atractylodes lancea* (Thunb.) Dc. (Cang Zhu), 10 in achyranthis bidentatae radix (Niu Xi), and 3 in Coicis Semen (Yi Yi Ren) in BATMAN-TCM.

#### 3.1.2. Selection of Compounds Using ADME Screening and Related Targets

All the identified compounds were selected through ADME screening, with 90 of 479 compounds satisfying the suggested criteria OB ≥ 30 and DL ≥ 0.18 [18–20]. Of the 90 compounds, 25 were duplicated and removed, and the remaining 65 compounds were included for further study. Moreover, of the excluded compounds that do not meet the suggested criteria, 7 compounds, including ferulic acid, beta-elemene, eugenol, and paeonol in Phellodendri Chinensis Cortex (Huang Bo) and geniposide, rutin, and astragalin in achyranthis bidentatae radix (Niu Xi), are considered bioactive compounds and included for further analysis, and the effects of ferulic acid [22], beta-elemene [23], eugenol [24–26], paeonol [27–29], geniposide [30–32], rutin [33, 34], and astragalin [35] on RA have been investigated. The final 72 compounds are selected from the four herbal medicines (Table 1). A total of 386 target genes related to the final identified compounds are obtained from the UniProt databases.

#### 3.1.3. Identified Disease Target Genes

The target genes related to RA were searched in GeneCards and OMIM databases, which include 3768 genes in GeneCards and 1 gene in OMIM, with no overlapping target gene.

#### 3.1.4. Intersection of Identified Targets of Compounds and Disease

**Figure 2:**
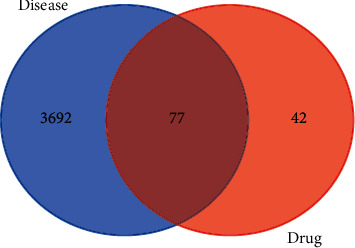
A Venn diagram showing intersection identified targets of identified compounds and RA.

In the Venn diagram intersection of identified targets about identified compounds and of RA (Figure 2), a total of 77 target genes are acquired.

### 3.2. Topological and PPI Network

#### 3.2.1. Topological Network Analysis

**Figure 3:**
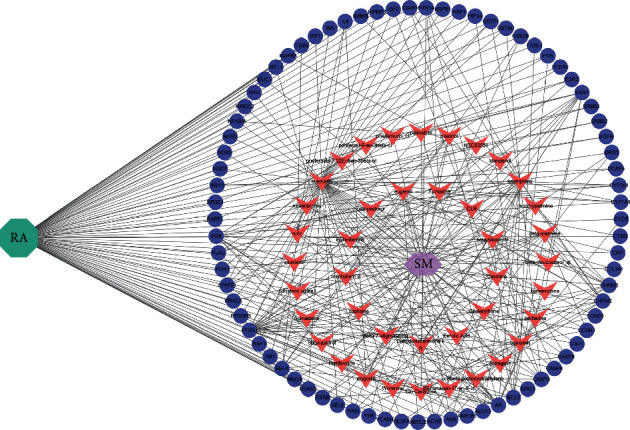
The SM-RA mechanism network. The green octagon represents rheumatoid arthritis (RA), the purple hexagon represents the herbal medicine simiao pill (SM), while pink V's represent compounds, and blue-purple ellipses represent genes.

**Figure 4:**
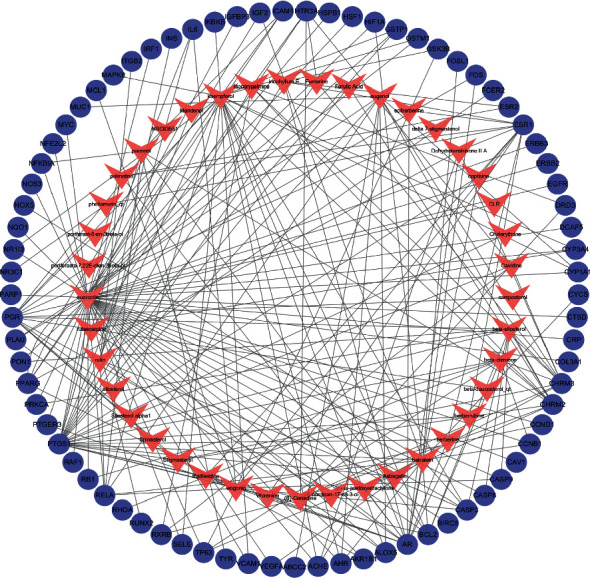
The compounds-targets network. The pink V's represent compounds and blue-purple ellipses represent target genes.

The SM-RA mechanism network (Figure 3) consists of 77 target genes nodes (shared gene of SM and RA), 41 compound nodes, and 349 edges. Among the 14 compounds (Dehydrotanshinone II A, Stigmasterol, beta-sitosterol, Isocorypalmine, beta-elemene, quercetin, eugenol, paeonol, (S)-Canadine, wogonin, baicalein, Inophyllum *E*, rutin, and kaempferol) that connected to more than four genes, 55 target genes are associated with quercetin, 22 target genes are associated with kaempferol, 15 target genes are associated with baicalein, 14 target genes are associated with wogonin, and 10 target genes are associated with beta-sitosterol and eugenol, respectively (Table 2). In addition, 10 genes, including PTGS1, ESR1, AR, PGR, CHRM3, PPARG, CHRM2, BCL2, CASP3, and RELA, are related to more than five compounds, as shown in the compounds-targets network (Figure 4). These compounds and genes may be the key nodes in the network.

#### 3.2.2. PPI Network

**Figure 5:**
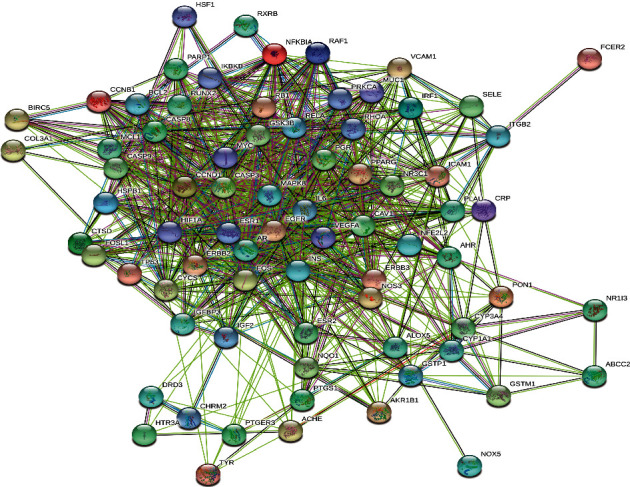
The PPI network of SM-RA. Each node represents the relevant gene, and the edges represent protein-protein associations.

**Figure 6:**
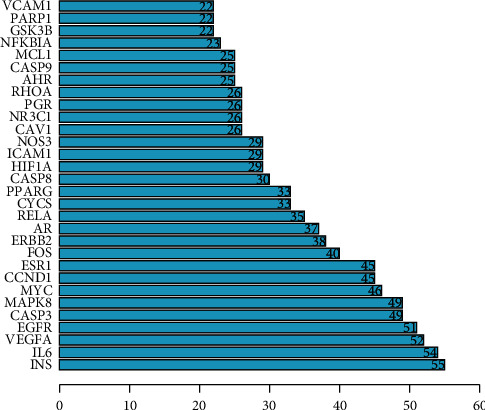
Hub top 30 genes of the PPI network. The *y*-axis displays significant top 30 genes, and the *x-*axis shows line counts of these genes.

The PPI network is established by setting the confidence level of more than 0.40 and hiding the independent target protein nodes. The PPI network nodes represent proteins and edges represent protein-protein interactions. The network has 75 nodes and 1604 edges (Figure 5). In addition, we analyzed the importance prioritization (adjacent nodes count of each protein) of proteins according to the network, and the leading 30 genes with higher connection were visualized by constructing a bar plot diagram (Figure 6), which indicates the 30 genes or proteins that may play a bridge role in connecting other nodes in the PPI network. These 30 genes or proteins include inflammation-associated genes (IL6 [36], NFKBIA [37]), cell proliferation-, differentiation-, and transformation-related genes (FOS [38], EGFR [39], MAPK8 [40], NR3C1 [41], RHOA [42], and PARP1 [43]), cell apoptosis-related genes (CASP3, CASP8 [44], CASP9 [45], MYC [46], CYCS [47], HIF1A [48], MCL1 [49], and GSK3B [50]), cell cycle-related gene (CCND1 [51]), hormone-related genes (INS [52], ESR1 [53], AR [54], and PGR [55]), angiogenesis-related gene (VEGFA [56]), and transcription factor (RELA [57]).

### 3.3. Enrichment Analysis

#### 3.3.1. GO Enrichment Analysis

**Figure 7:**
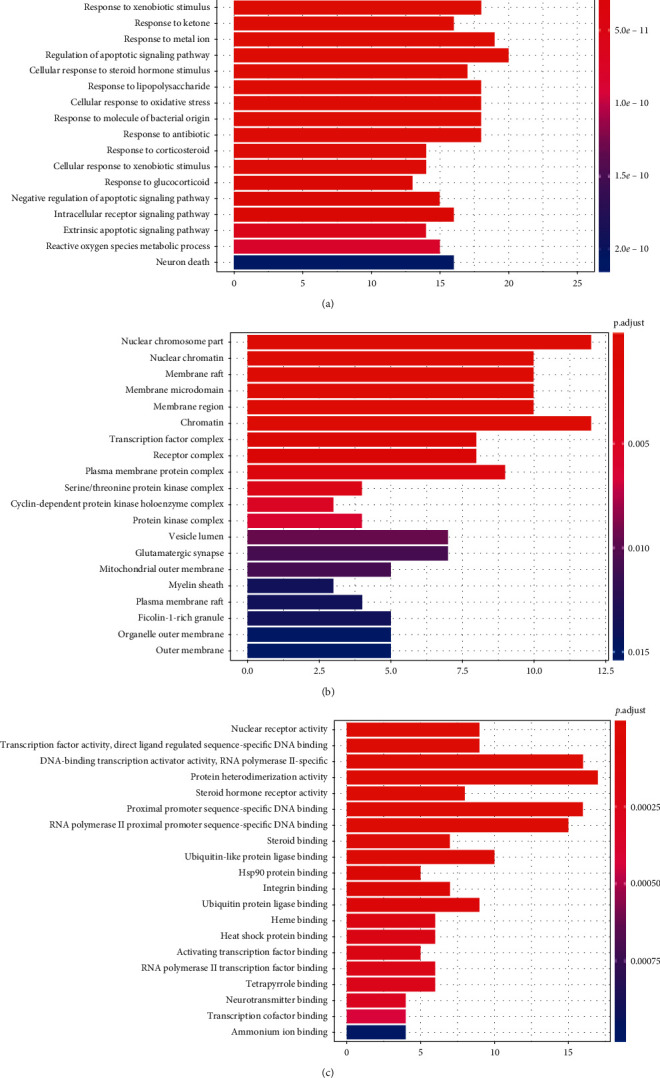
GO analysis of targets, the top 20 significant enrichment terms in BP (a), CC (b), and MF (c). The *y*-axis shows significantly enriched biological process, cellular component, and molecular function categories of the target genes, respectively. The redder the color, the lower the *P* value. The *x*-axis displays the enrichment scores of these terms, and the length of the bar indicates the number of target genes in each pathway.

GO analysis consisted of biological process (BP), cellular component (CC), and molecular function (MF). As showed in Figure 7, the top 20 enrichment terms are visualized by the bar plot diagram. The results demonstrated that numerous targets are involved in various BPs associated with immune response and inflammation, such as the response to a steroid hormone, response to oxidative stress, and regulation of the apoptotic signaling pathway, which confirmed strongly the correlation with the pathogenesis in RA. The CC results showed that most of the targets are localized to the cellular membrane and nuclear chromatin part. The MF results indicated that many targets are associated with nuclear receptor activity and transcription factor activity.

#### 3.3.2. KEGG Enrichment Analysis

**Figure 8:**
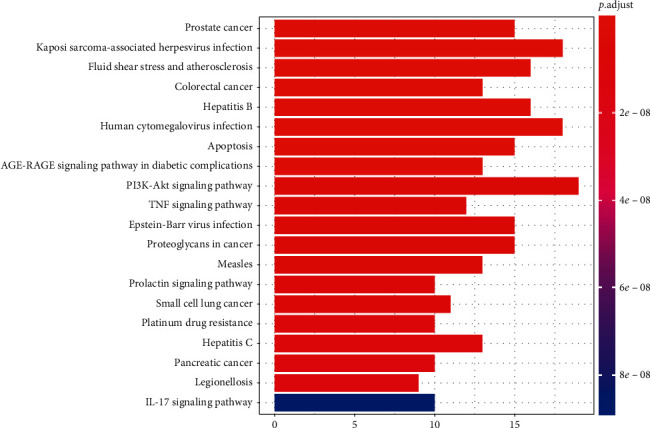
KEGG enrichment pathways (top 20). The *y*-axis displays the top 20 significantly enriched KEGG pathways of the target genes. The redder the color, the smaller the *P* value. The *x*-axis represents the target genes counts, and the length of the bar indicates the number of target genes in each pathway.

**Figure 9:**
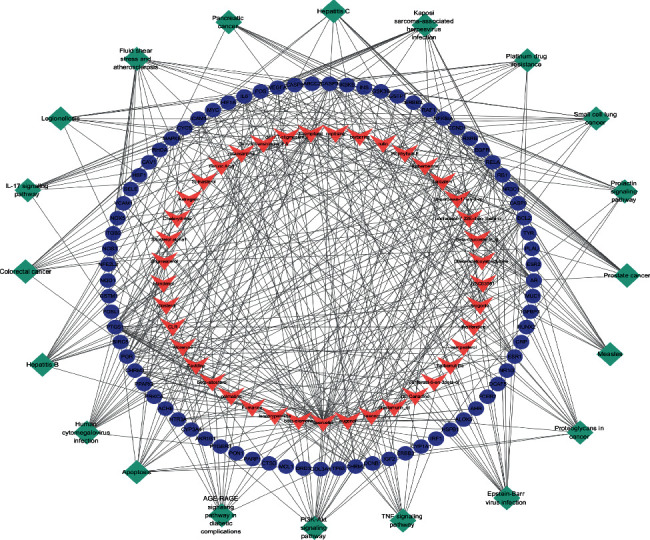
The pathways-targets-compounds network. The green diamonds represent pathways, the blue-purple ellipses represent genes, and the pink V's represent compounds.

The KEGG pathways are applied to examine the function and signaling pathways of the identified target genes, with the top 20 of the potential pathways (*P* < 0.05 and *q* < 0.05) shown by a bar plot diagram (Figure 8) and visualized with the pathways-targets-compounds network (Figure 9). The results showed that numerous targets are associated with certain virus infections (such as Epstein-Barr virus infection) and cancer, which are associated with the onset and prognosis of RA.

### 3.4. Molecular Docking Analysis

**Figure 10:**
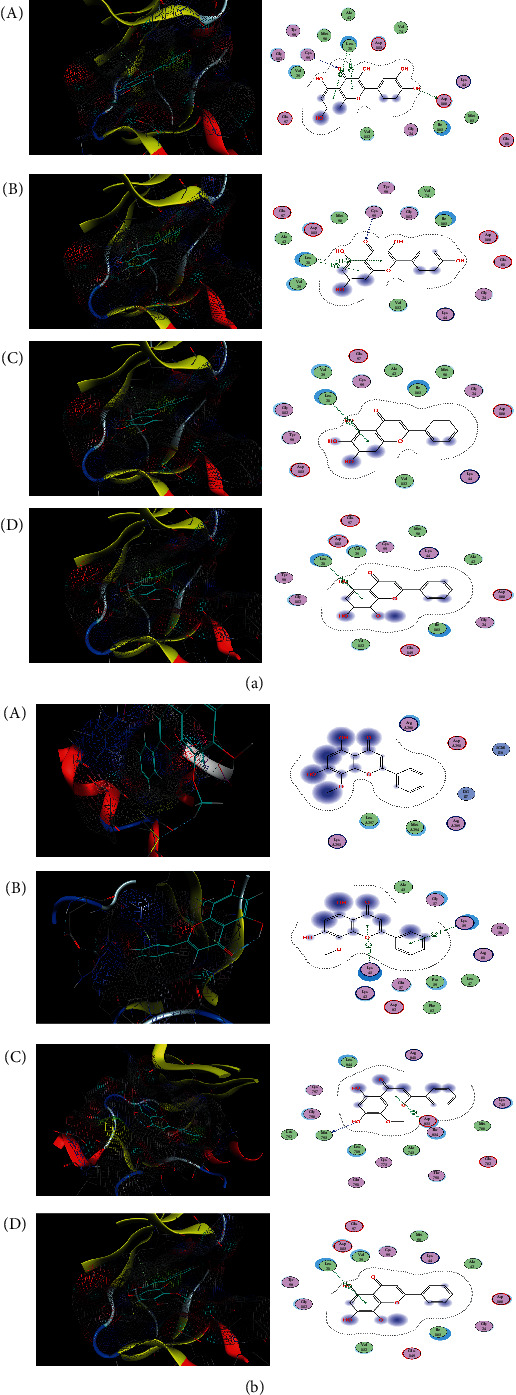
Molecular docking results. (a) The action mode of NFKBIA and quercetin, kaempferol, baicalein, and wogonin: (A) NFKBIA and quercetin; (B) NFKBIA and kaempferol; (C) NFKBIA and baicalein; (D) NFKBIA and wogonin. (b) The action mode of wogonin and IL6, VEGFA, EGFR, and NFKBIA: (A) wogonin and IL6; (B) wogonin and VEGFA; (C) wogonin and EGFR; (D) wogonin and NFKBIA.

The selected targets, including IL6, VEGFA, EGFR, and NFKBIA, play a significant role in the SM-RA network. The candidate compounds, including quercetin, kaempferol, baicalein, and wogonin, are the top 4 compounds (ranking by related target genes count) in the SM-RA network. These 4 target genes and 4 compounds are imported into MOE for molecular docking verification. The docking scores are shown in Table 3. The action mode of NFKBIA and quercetin, kaempferol, baicalein, and wogonin and the action mode of wogonin and IL6, VEGFA, EGFR, and NFKBIA are shown in Figure 10.

## 4. Discussion

In the present network pharmacological analysis, a total of 479 compounds were identified in the four herbal medicines of SM, and 72 compounds were yielded by ADME criteria screening. A total of 386 targets related to potential compounds and 3769 targets associated with RA were identified, and 77 target genes were obtained from the interaction of targets about SM identified compounds and RA. SM-RA network analysis visualized the interaction of multicomponents and multitargets about SM on RA. The compounds-targets network analysis indicated that the 6 compounds, including quercetin, kaempferol, baicalein, wogonin, beta-sitosterol, and eugenol, were linked to ≥10 target genes, and the 10 target genes (PTGS1, ESR1, AR, PGR, CHRM3, PPARG, CHRM2, BCL2, CASP3, and RELA) were core target genes in the network. GO enrichment analysis indicated that numerous targets are involved in response to a steroid hormone, oxidative stress, and regulation of the apoptotic signaling pathway in BP, are localized to the cellular membrane and nuclear chromatin part in CC, and are associated with nuclear receptor activity and transcription factor activity in MF. KEGG pathways analysis results indicated that numerous targets are associated with certain virus infections and cancer. Molecular docking showed that quercetin, kaempferol, baicalein, and wogonin have good binding activity with IL6, VEGFA, EGFR, and NFKBIA targets.

About 72 identified compounds, particularly the 6 compounds, including quercetin, kaempferol, baicalein, wogonin, beta-sitosterol, and eugenol, were linked to more than 10 targets, indicating that these compounds might play a vital role in the process of RA treatment. Furthermore, certain compounds have exhibited the potential antirheumatic therapeutic activities except for wogonin (Table 4). For instance, quercetin has been reported to decrease levels of tumor necrosis factor-*α* (TNF-*α*), interleukin-1*β* (IL-1*β*), interleukin-17 (IL-17), and monocyte chemotactic protein-1 (MCP-1) [58] and significantly reduced damage to interchondral joints, infiltration of inflammatory cells, and pannus formation [59]. Besides, kaempferol suppresses the proliferation and migration of RAFLS and the release of activated T-cell-mediated inflammatory cytokines and reduces osteoclast differentiation through targeting on the fibroblast growth factor receptor 3- (FGFR3-) ribosomal S6 kinase 2 (RSK2) signaling axis [60]. In addition, baicalein inhibits human rheumatoid arthritis fibroblast-like synoviocytes (RAFLS) proliferation involving suppression of nuclear factor kappa B (NF-*κ*B) transcriptional activity and recombinant macrophage migration inhibitory factor- (MIF-) mediated signaling [61]. What's more, *β*-Sitosterol could modulate the functions of macrophages and attenuates rheumatoid inflammation in CIA mice [62]. For eugenol, it is reported to be effective in ameliorating oxidative stress and inflammation in arthritic rats [25, 26]. Moreover, among the other 66 compounds, some articles previously reported the antirheumatic effect. For example, ferulic acid is reported to suppress osteoclast differentiation and bone erosion via the inhibition of receptor activator of nuclear factor кB ligand- (RANKL-) dependent NF-*κ*B signaling pathway [63], and berberine could attenuate adjuvant-induced arthritic fibroblast-like synoviocytes (AA-FLS) proliferation and regulate the Th17/Treg imbalance [64]. Collectively, these active components exhibit antirheumatic effects from various aspects, including anti-inflammatory, immunoregulatory, reducing bone erosion and destruction, and attenuating oxidative stress. Therefore, these might indicate the collective effectiveness and diversity of constituents in SM for treating RA.

Among the main target genes (top 30) in the PPI network is INS, ranking first with the highest connection, which may affect the local inflammatory process of joint in RA [52], though research about the role of INS in RA is rare. IL6 is involved in the regulation of the immune response, inflammation, and hematopoiesis and confirmed the pathological roles in RA [65]. VEGFA contributes to promoting the angiogenic phenotype of RA [56]. EGFR is proved to be involved in the proliferation and cytokine production of synovial fibroblasts, the proliferation of endothelial cells, and the formation of osteoclasts [39]. CASP3, CASP8 [44], and CASP9 [45] are involved in the apoptosis of RA synoviocytes. NFKBIA is related to the inflammation of RA by regulating many genes for immune response, cell adhesion, differentiation, proliferation, angiogenesis, and apoptosis [37]. The antirheumatic effect of aforementioned baicalein, ferulic acid, etc. is partially associated with these target genes, indicating the interaction between multicomponents and multitargets of SM treating RA.

KEGG pathway enrichment analysis indicated that certain types of virus infection and cancer might also be crucial in the network. The evidence that viral infection contributes to RA, such as Epstein-Barr virus infection [1], is strong, and RA is associated with an increased risk of cancer [66]. In addition, the KEGG pathway analysis also indicated that PI3K-Akt, TNF, and IL-17 signaling pathway may be a critical signaling pathway in the network pharmacology. The PI3K-Akt signaling pathway is involved in inflammatory cytokine production [67], proliferation and migration of RAFLS [68] and chondrocyte proliferation [69], and apoptosis and autophagy in RA [69]. Moreover, a pivotal role for the proinflammatory cytokines, including tumor necrosis factor (TNF) [70] and interleukin- (IL-) 17 [71], in RA joint pathology has been identified.

Of the leading 30 target genes with a higher connection in the PPI network, IL6, VEGFA, EGFR, and NFKBIA play a critical role in the development of RA, which has been aforementioned. Besides, in the visualized pathways-targets network, IL6, VEGFA, EGFR, and NFKBIA are involved in numerous pathways, indicating that SM may exert anti-RA effects through multipathways and multitargets combined interaction. Furthermore, the molecular docking analysis was constructed to investigate the interaction of some candidate compounds and targets. For example, the absolute value of docking scores about NFKBIA and quercetin, kaempferol, baicalein, and wogonin is the highest in each group, indicating that NFKBIA has a higher binding affinity than other target genes. For wogonin, although there have been no relevant studies about the effect in RA, the docking results indicated that wogonin performed good binding activity with IL6, VEGFA, EGFR, and NFKBIA. In brief, the high binding affinities of these active components indicated that the therapeutic effects of SM treating RA were probably through the modulation of several related targets.

As shown, the anti-RA effect of identified compounds (quercetin, kaempferol, baicalein, beta-sitosterol, and eugenol) is partially associated with the potential target genes, including NFKBIA, IL6, and MAPK, and potential signals, including PI3K-Akt, TNF, and IL-17, indicating the interaction between multicomponents, multitargets, and multisignaling of SM treating RA.

## 5. Limitation

This study has some limitations. It provides only a predictive overview of the pharmacological mechanisms of SM against RA based on the existing database, and further experiment verification in vivo and in vitro is necessary to ensure the reliability and reasonability of predicted results. First, posttranscriptional processing, translation regulation, and posttranslational processing and regulation play a critical role in gene expression regulation, and most of the research on mechanisms about SM treating RA is gene level in this study; therefore, an in-depth study needs to explore the related mechanism. Second, the key proteins and KEGG pathways need to be verified. Third, the anti-RA effect needs to be further verified in the animal model.

Besides, for clinical applying of SM treating RA, owing to the ethnic, genetic, and possible etiological differences, potential mechanisms about the related therapeutic module coinciding with clinical applications are worthy of further experimental investigation. In addition, importantly, dosage exploration, oral bioavailability, water-solubility, pharmacokinetics, and potential side effects of SM will also need a thorough exploration.

## 6. Conclusion

In summary, a bioinformatics/topology-based strategy, including ADEM screening, bioinformatics, network topology, enrichment analysis, and molecular analysis, was applied for identification of the molecular mechanisms of SM against RA. The integrated strategy might make the decipherment of biological mechanisms more accurate and efficient. The SM-RA network, compounds-targets network, and pathways-targets network analysis visualized the interaction of multicomponents and multitargets about SM treating RA. In particular, quercetin, kaempferol, baicalein, wogonin, beta-sitosterol, and eugenol might be the candidate therapeutic agents, and PTGS1, ESR1, AR, PGR, CHRM3, PPARG, CHRM2, BCL2, CASP3, and RELA were identified as potential drug targets. The enrichment and PPI analysis revealed the biological functions of the grouping networks related to the pathogenesis of RA. The multicomponent cosynergism of the herbal combinations about SM was elaborated. The study also revealed the multifunctional synergetic mechanisms of SM, including certain virus infection and cancer, and PI3K-Akt, TNF, and IL-17 signaling pathway.

## Figures and Tables

**Table 1:** 72 active compounds of SM.

Mol id	Molecule name	OB (%)	DL	Herb
MOL002636	Kihadalactone A	34.21	0.82	Phellodendri Chinensis Cortex
MOL013352	Obacunone	43.29	0.77	Phellodendri Chinensis Cortex
MOL002641	Phellavin_qt	35.86	0.44	Phellodendri Chinensis Cortex
MOL002644	Phellopterin	40.19	0.28	Phellodendri Chinensis Cortex
MOL002651	Dehydrotanshinone II A	43.76	0.40	Phellodendri Chinensis Cortex
MOL002652	delta7-dehydrosophoramine	54.45	0.25	Phellodendri Chinensis Cortex
MOL002656	Dihydroniloticin	36.43	0.81	Phellodendri Chinensis Cortex
MOL002659	Kihadanin A	31.60	0.70	Phellodendri Chinensis Cortex
MOL002660	Niloticin	41.41	0.82	Phellodendri Chinensis Cortex
MOL002662	Rutaecarpine	40.30	0.60	Phellodendri Chinensis Cortex
MOL002663	Skimmianin	40.14	0.20	Phellodendri Chinensis Cortex
MOL002666	Chelerythrine	34.18	0.78	Phellodendri Chinensis Cortex
MOL002668	Worenine	45.83	0.87	Phellodendri Chinensis Cortex
MOL002670	Cavidine	35.64	0.81	Phellodendri Chinensis Cortex
MOL002671	Candletoxin A	31.81	0.69	Phellodendri Chinensis Cortex
MOL002672	Hericenone H	39.00	0.63	Phellodendri Chinensis Cortex
MOL002673	Hispidone	36.18	0.83	Phellodendri Chinensis Cortex
MOL000358	Beta-sitosterol	36.91	0.75	Phellodendri Chinensis Cortex
MOL000622	Magnograndiolide	63.71	0.19	Phellodendri Chinensis Cortex
MOL000762	Palmidin A	35.36	0.65	Phellodendri Chinensis Cortex
MOL000787	Fumarine	59.26	0.83	Phellodendri Chinensis Cortex
MOL000790	Isocorypalmine	35.77	0.59	Phellodendri Chinensis Cortex
MOL001131	phellamurin_qt	56.60	0.39	Phellodendri Chinensis Cortex
MOL001455	(S)-canadine	53.83	0.77	Phellodendri Chinensis Cortex
MOL001771	Poriferast-5-en-3beta-ol	36.91	0.75	Phellodendri Chinensis Cortex
MOL002894	Berberrubine	35.74	0.73	Phellodendri Chinensis Cortex
MOL005438	Campesterol	37.58	0.71	Phellodendri Chinensis Cortex
MOL006392	Dihydroniloticin	36.43	0.82	Phellodendri Chinensis Cortex
MOL006401	Melianone	40.53	0.78	Phellodendri Chinensis Cortex
MOL006413	Phellochin	35.41	0.82	Phellodendri Chinensis Cortex
MOL006422	Thalifendine	44.41	0.73	Phellodendri Chinensis Cortex
MOL002665	Ferulic acid	40.43	0.06	Phellodendri Chinensis Cortex
MOL000908	Beta-elemene	25.63	0.06	Phellodendri Chinensis Cortex
MOL000254	Eugenol	56.24	0.04	Phellodendri Chinensis Cortex
MOL000874	Paeonol	28.79	0.04	Phellodendri Chinensis Cortex
MOL000179	2-Hydroxyisoxypropyl-3-hydroxy-7-isopentene-2,3-dihydrobenzofuran-5-carboxylic	45.20	0.20	*Atractylodes lancea* (Thunb.) Dc.
MOL000184	NSC63551	39.25	0.76	*Atractylodes lancea* (Thunb.) Dc.
MOL000186	Stigmasterol 3-O-beta-D-glucopyranoside_qt	43.83	0.76	*Atractylodes lancea* (Thunb.) Dc.
MOL000188	3*β*-acetoxyatractylone	40.57	0.22	*Atractylodes lancea* (Thunb.) Dc.
MOL000088	Beta-sitosterol 3-O-glucoside_qt	36.91	0.75	*Atractylodes lancea* (Thunb.) Dc.
MOL000092	daucosterin_qt	36.91	0.76	*Atractylodes lancea* (Thunb.) Dc.
MOL000094	daucosterol_qt	36.91	0.76	*Atractylodes lancea* (Thunb.) Dc.
MOL001006	Poriferasta-7,22E-dien-3beta-ol	42.98	0.76	Achyranthis bidentatae radix
MOL012461	28-Norolean-17-en-3-ol	35.93	0.78	Achyranthis bidentatae radix
MOL012505	bidentatoside, ii_qt	31.76	0.59	Achyranthis bidentatae radix
MOL012537	Spinoside A	41.75	0.40	Achyranthis bidentatae radix
MOL012542	*β*-ecdysterone	44.23	0.82	Achyranthis bidentatae radix
MOL002714	Baicalein	33.52	0.21	Achyranthis bidentatae radix
MOL002776	Baicalin	40.12	0.75	Achyranthis bidentatae radix
MOL002897	Epiberberine	43.09	0.78	Achyranthis bidentatae radix
MOL003847	Inophyllum E	38.81	0.85	Achyranthis bidentatae radix
MOL000422	Kaempferol	41.88	0.24	Achyranthis bidentatae radix
MOL004355	Spinasterol	42.98	0.76	Achyranthis bidentatae radix
MOL012516	Geniposide	8.40	0.44	Achyranthis bidentatae radix
MOL000415	Rutin	3.20	0.68	Achyranthis bidentatae radix
MOL000561	Astragalin	14.03	0.74	Achyranthis bidentatae radix
MOL001323	Sitosterol alpha1	43.28	0.78	Coicis Semen
MOL001494	Mandenol	42.00	0.19	Coicis Semen
MOL002372	(6 *Z*, 10 *E*, 14 *E*, 18 *E*)-2,6,10,15,19,23-hexamethyltetracosa-2,6,10,14,18,22-hexaene	33.55	0.42	Coicis Semen
MOL002882	[(2R)-2,3-dihydroxypropyl] (Z)-octadec-9-enoate	34.13	0.30	Coicis Semen
MOL000359	Sitosterol	36.91	0.75	Coicis Semen
MOL008118	Coixenolide	32.40	0.43	Coicis Semen
MOL008121	2-Monoolein	34.23	0.29	Coicis Semen
MOL000953	CLR	37.87	0.68	Coicis Semen
MOL001454	Berberine	36.86	0.78	Phellodendri Chinensis Cortex, achyranthis bidentatae radix
MOL001458	Coptisine	30.67	0.86	Phellodendri Chinensis Cortex, achyranthis bidentatae radix
MOL002643	Delta 7-stigmastenol	37.42	0.75	Phellodendri Chinensis Cortex, achyranthis bidentatae radix
MOL000785	Palmatine	64.60	0.65	Phellodendri Chinensis Cortex, achyranthis bidentatae radix
MOL000098	Quercetin	46.43	0.28	Phellodendri Chinensis Cortex, achyranthis bidentatae radix
MOL000173	Wogonin	30.68	0.23	*Atractylodes lancea* (Thunb.) Dc. Achyranthis bidentatae radix
MOL000085	Beta-daucosterol_qt	36.91	0.75	*Atractylodes lancea* (Thunb.) Dc. Achyranthis bidentatae radix
MOL000449	Stigmasterol	43.83	0.76	Phellodendri Chinensis Cortex, achyranthis bidentatae radix, Coicis Semen

**Table 2:** Target genes interacting with compounds in the SM-RA network.

Compounds	Target genes
Delta 7-stigmastenol	PGR
Poriferast-5-en-3beta-ol	PGR
Campesterol	PGR
NSC63551	PGR
Beta-daucosterol_qt	PGR
Poriferasta-7,22E-dien-3beta-ol	PGR
28-Norolean-17-en-3-ol	PGR
Spinasterol	PGR
Sitosterol alpha1	PGR
Sitosterol	PGR
CLR	PGR
Chelerythrine	PTGS1
Astragalin	PTGS1
Mandenol	PTGS1
Ferulic acid	PTGS1, CHRM2
phellamurin_qt	ESR1, NR3C1
Epiberberine	ESR1, AR
Berberine	PTGS1, ESR1, AR
Coptisine	PTGS1, ESR1, AR
Worenine	PTGS1, ESR1, AR
Berberrubine	PTGS1, ESR1, AR
Thalifendine	PTGS1, ESR1, AR
Fumarine	PTGS1, CHRM3, HTR3A
Rutaecarpine	PTGS1, AR, HTR3A, CYP3A4
Cavidine	PTGS1, CHRM3, HTR3A, RXRB
Palmatine	PTGS1, ESR1, AR, ESR2
3*β*-acetoxyatractylone	CHRM3, AR, ACHE, CHRM2
Dehydrotanshinone II A	CHRM3, ESR1, AR, PPARG, ACHE
Inophyllum E	PTGS1, ESR1, AR, ESR2, GSK3B
Stigmasterol	PGR, PTGS1, AKR1B1, PLAU, CHRM3, CHRM2
Isocorypalmine	PTGS1, CHRM3, HTR3A, CHRM2, DRD3, RXRB
(S)-canadine	PTGS1, CHRM3, HTR3A, CHRM2, DRD3, RXRB
Paeonol	PTGS1, CHRM2, RELA, BCL2, NFKBIA, ICAM1, TYR
Beta-elemene	CHRM2, PTGS1, CHRM3, BCL2, RB1, TP63, CCNB1, RHOA
Rutin	RELA, IL6, CASP3, ALOX5, GSTP1, INS, FCER2, ITGB2
Beta-sitosterol	PGR, PTGS1, CHRM3, CHRM2, BCL2, CASP9, CASP3, CASP8, PRKCA, PON1
Eugenol	PTGS1, CHRM3, CHRM2, PLAU, RELA, CYP1A1, ALOX5, AHR, ABCC2, MUC1
Wogonin	PTGS1, ESR1, AR, PPARG, GSK3B, RELA, CCND1, BCL2, CASP9, IL6, CASP3, TP63, PTGER3, MCL1
Baicalein	PTGS1, AR, RELA, VEGFA, BCL2, FOS, CASP3, TP63, HIF1A, FOSL1, CCNB1, AHR, IGF2, CYCS, NOX5
Kaempferol	PTGS1, AR, PPARG, PGR, ACHE, CHRM2, RELA, IKBKB, BCL2, CASP3, MAPK8, PPARG, CYP3A4, CYP1A1, ICAM1, SELE, VCAM1, ALOX5, GSTP1, AHR, NR1I3, GSTM1
Quercetin	PTGS1, AR, PPARG, AKR1B1, ACHE, RELA, EGFR, VEGFA, CCND1, BCL2, FOS, CASP9, PLAU, RB1, IL6, CASP3, TP63, NFKBIA, CASP8,RAF1, PRKCA, HIF1A,ERBB2,PPARG,CYP3A4,CAV1,MYC,CYP1A1, ICAM1, SELE, VCAM1, PTGER3, BIRC5, NOS3, HSPB1, CCNB1, ALOX5, GSTP1, NFE2L2, NQO1, PARP1, AHR, COL3A1, DCAF5, NR1I3, HSF1, CRP, RUNX2, CTSD, IGFBP3, IGF2, IRF1, ERBB3, PON1, GSTM1

**Table 3:** Molecular docking scores.

	IL6	VEGFA	EGFR	NFKBIA
Quercetin	−4.7051	−5.9131	−6.0857	−6.7291
Kaempferol	−4.9898	−5.4844	−5.7466	−6.5524
Baicalein	−4.5010	−5.5058	−5.8372	−6.2222
Wogonin	−4.6678	−5.6466	−6.1084	−6.6169

**Table 4:** Potential anti-RA mechanisms of some compounds.

Compound	Mechanism	Model	Reference
Quercetin	Decreased TNF-*α*, IL-1*β*, IL-17, and MCP-1	CIA mice	Haleagrahara et al. [58]
	Decreased TNF-*α* in joints, reduced interchondral joints damage, inflammatory cells infiltration, and pannus formation	CIA mice	Kawaguchi et al. [59]
	Promote RAFLS apoptosis by upregulating lncRNA metastasis-associated lung adenocarcinoma transcript 1 (MALAT1) and inhibiting PI3K/AKT signal activation subsequently	RAFLS	Pan et al. [72]
	Exerted anti-inflammatory, analgesic, and antioxidant effects by inhibiting NF-*κ*B and regulating nuclear factor erythroid 2-related factor (Nrf2)/home oxygenase (HO-1) signal	Zymosan-induced arthritis mice	Guazelli, et al. [73]
	Inhibited IL-17 and RANKL production, suppressed Th17 cell	RAFLS	Kim HR, et al. 2019 [74]

Kaempferol	Inhibited RAFLS proliferation and migration, suppressed inflammatory cytokines (IL-17, IL-21, and TNF-*α*) by targeting FGFR3-RSK2 signal	RAFLS	Lee, et al. [60]
	Inhibited RAFLS migration and invasion by blocking MAPK signal	RAFLS	Pan et al. [75]
	Inhibited RAFLS proliferation, reduced MMPs, COX-2, and PGE2 production, inhibited NF-*κ*B activation	RAFLS	Yoon et al. [76]
Baicalein	Inhibited RAFLS proliferation by suppressing NF-*κ*B activation	RAFLS	Chen et al. [61]

Beta-sitosterol	Inhibited inflammatory cytokines (iNOS, IL-1*β*), modulated macrophages functions	CIA mice	Liu et al. [62]

Eugenol	Inhibited mononuclear infiltration, lowered TNF-*α*, TGF-*β*, and IFN-*γ*	CIA murine	Grespan et al. [24]
	Reduced inflammatory cytokines (TNF-*α*, IL-6, and IL-10) and oxidative stress	CIA rat	Mateen et al. [25]
	Reduced inflammatory cytokines (TNF-*α*, IL-6) and oxidative stress	RA patients	Mateen et al. [26]

The anti-RA effect of identified compounds (quercetin, kaempferol, baicalein, beta-sitosterol, and eugenol) is partially associated with the potential target genes, including NFKBIA, IL6, and MAPK, and potential signals, including PI3K‐AKT, TNF, and IL‐17, indicating the interaction between multicomponents, multitargets, and multisignaling of SM treating RA.

## Data Availability

The figures and tables used to support the findings of this study are included within the article, and the original data are available from the first author or corresponding author upon request.

## References

[1] Aletaha D., Smolen J. S. (2018). Diagnosis and management of rheumatoid arthritis. *JAMA*.

[2] Tanaka Y., Takeuchi T., Tanaka S. (2019). Efficacy and safety of peficitinib (ASP015K) in patients with rheumatoid arthritis and an inadequate response to conventional DMARDs: a randomised, double-blind, placebo-controlled phase III trial (RAJ3). *Annals of the Rheumatic Diseases*.

[3] Scott D. L., Wolfe F., Huizinga T. W. (2010). Rheumatoid arthritis. *The Lancet*.

[4] McInnes I. B., Schett G. (2017). Pathogenetic insights from the treatment of rheumatoid arthritis. *The Lancet*.

[5] Burmester G. R., Pope J. E. (2017). Novel treatment strategies in rheumatoid arthritis. *The Lancet*.

[6] Moudgil K. D., Berman B. M. (2014). Traditional Chinese medicine: potential for clinical treatment of rheumatoid arthritis. *Expert Review of Clinical Immunology*.

[7] Seca S., Franconi G. (2018). Understanding Chinese medicine patterns of rheumatoid arthritis and related biomarkers. *Medicines (Basel)*.

[8] Yuan H.-Y., Zhang X.-L., Zhang X.-H., Meng L., Wei J.-F. (2015). Analysis of patents on anti-rheumatoid arthritis therapies issued in China. *Expert Opinion on Therapeutic Patents*.

[9] Lü S., Wang Q., Li G., Sun S., Guo Y., Kuang H. (2015). The treatment of rheumatoid arthritis using Chinese medicinal plants: from pharmacology to potential molecular mechanisms. *Journal of Ethnopharmacology*.

[10] Liu Y. F., Huang Y., Wen C. Y. (2017). The effects of modified simiao decoction in the treatment of gouty arthritis: a systematic review and meta-analysis. *Evidence Based Complementary and Alternative Medicine*.

[11] Ma C.-H., Kang L.-L., Ren H.-M., Zhang D.-M., Kong L.-D. (2015). Simiao pill ameliorates renal glomerular injury via increasing Sirt1 expression and suppressing NF-*κ*B/NLRP3 inflammasome activation in high fructose-fed rats. *Journal of Ethnopharmacology*.

[12] Shen P., Tu S., Wang H., Qin K., Chen Z. (2019). Simiao pill attenuates collagen-induced arthritis in rats through suppressing the ATX-LPA and MAPK signalling pathways. *Evidence Based Complementary and Alternative Medicine*.

[13] Zhao J., Zha Q., Jiang M., Cao H., Lu A. (2013). Expert consensus on the treatment of rheumatoid arthritis with Chinese patent medicines. *The Journal of Alternative and Complementary Medicine*.

[14] Li S., Zhang B. (2013). Traditional Chinese medicine network pharmacology: theory, methodology and application. *Chinese Journal of Natural Medicines*.

[15] Hopkins A. L. (2008). Network pharmacology: the next paradigm in drug discovery. *Nature Chemical Biology*.

[16] Luo T. T., Lu Y., Yan S. K. (2019). Network pharmacology in research of Chinese medicine formula: methodology, application and prospective. *Chinese Journal of Integrative Medicine*.

[17] Guo Q., Zheng K., Fan D. (2017). Wu-Tou Decoction in Rheumatoid arthritis: integrating network pharmacology and in vivo pharmacological evaluation. *Frontiers in Pharmacology*.

[18] Lee A. Y., Park W., Kang T.-W., Cha M. H., Chun J. M. (2018). Network pharmacology-based prediction of active compounds and molecular targets in Yijin-Tang acting on hyperlipidaemia and atherosclerosis. *Journal of Ethnopharmacology*.

[19] Xie G., Peng W., Li P. (2018). A network pharmacology analysis to explore the effect of astragali radix-radix angelica sinensis on traumatic brain injury. *Biomed Research International*.

[20] Li P., Chen J., Zhang W., Li H., Wang W., Chen J. (2019). Network pharmacology based investigation of the effects of herbal ingredients on the immune dysfunction in heart disease. *Pharmacological Research*.

[21] Tsaioun K., Blaauboer B. J., Hartung T. (2016). Evidence-based absorption, distribution, metabolism, excretion (ADME) and its interplay with alternative toxicity methods. *Altex*.

[22] Ganesan R., Rasool M. (2018). Ferulic acid inhibits interleukin 17-dependent expression of nodal pathogenic mediators in fibroblast-like synoviocytes of rheumatoid arthritis. *Journal of Cellular Biochemistry*.

[23] Zou S., Wang C., Cui Z. (2016). *β*-Elemene induces apoptosis of human rheumatoid arthritis fibroblast-like synoviocytes via reactive oxygen species-dependent activation of p38 mitogen-activated protein kinase. *Pharmacological Reports*.

[24] Grespan R., Paludo M., Lemos H. d. P. (2012). Anti-arthritic effect of eugenol on collagen-induced arthritis experimental model. *Biological and Pharmaceutical Bulletin*.

[25] Mateen S., Shahzad S., Ahmad S. (2019). Cinnamaldehyde and eugenol attenuates collagen induced arthritis via reduction of free radicals and pro-inflammatory cytokines. *Phytomedicine*.

[26] Mateen S., Rehman M. T., Shahzad S. (2019). Anti-oxidant and anti-inflammatory effects of cinnamaldehyde and eugenol on mononuclear cells of rheumatoid arthritis patients. *European Journal of Pharmacology*.

[27] Li Y., Li P., Lin S.-H., Zheng Y.-Q., Zheng X.-X. (2014). Paeonol inhibited TNF-*α*-induced GM-CSF expression in fibroblast-like synoviocytes. *International Journal of Clinical Pharmacology and Therapeutics*.

[28] Liu N., Feng X., Wang W., Zhao X., Li X. (2017). Paeonol protects against TNF-*α*-induced proliferation and cytokine release of rheumatoid arthritis fibroblast-like synoviocytes by upregulating FOXO3 through inhibition of miR-155 expression. *Inflammation Research*.

[29] Zhai K. F., Duan H., Luo L. (2017). Protective effects of paeonol on inflammatory response in IL-1beta-induced human fibroblast-like synoviocytes and rheumatoid arthritis progression via modulating NF-kappaB pathway. *Inflammopharmacology*.

[30] Li F., Dai M., Wu H. (2018). Immunosuppressive effect of geniposide on mitogen-activated protein kinase signalling pathway and their cross-talk in fibroblast-like synoviocytes of adjuvant arthritis rats. *Molecules*.

[31] Deng R., Li F., Wu H. (2018). Anti-inflammatory mechanism of geniposide: inhibiting the hyperpermeability of fibroblast-like synoviocytes via the RhoA/p38MAPK/NF-kappaB/F-actin signal pathway. *Frontiers in Pharmacology*.

[32] Wang Y., Dai L., Wu H. (2018). Novel anti-inflammatory target of geniposide: inhibiting Itg*β*1/Ras-Erk1/2 signal pathway via the miRNA-124a in rheumatoid arthritis synovial fibroblasts. *International Immunopharmacology*.

[33] Sun C. L., Wei J., Bi L. Q. (2017). Rutin attenuates oxidative stress and proinflammatory cytokine level in adjuvant induced rheumatoid arthritis via inhibition of NF-kappaB. *Pharmacology*.

[34] Gul A., Kunwar B., Mazhar M. (2018). Rutin and rutin-conjugated gold nanoparticles ameliorate collagen-induced arthritis in rats through inhibition of NF-*κ*B and iNOS activation. *International Immunopharmacology*.

[35] Jia Q., Wang T., Wang X. (2019). Astragalin suppresses inflammatory responses and bone destruction in mice with collagen-induced arthritis and in human fibroblast-like synoviocytes. *Frontiers in Pharmacology*.

[36] Pandolfi F., Franza L., Carusi V. (2020). Interleukin-6 in rheumatoid arthritis. *International Journal of Molecular Science*.

[37] Sun X. F., Zhang H. (2007). NFKB and NFKBI polymorphisms in relation to susceptibility of tumour and other diseases. *Histology and Histopathology*.

[38] Hannemann N., Jordan J., Paul S. (2017). The AP-1 transcription factor c-jun promotes arthritis by regulating cyclooxygenase-2 and arginase-1 expression in macrophages. *The Journal of Immunology*.

[39] Swanson C. D., Akama-Garren E. H., Stein E. A. (2012). Inhibition of epidermal growth factor receptor tyrosine kinase ameliorates collagen-induced arthritis. *The Journal of Immunology*.

[40] Johnson G. L., Lapadat R. (2002). Mitogen-activated protein kinase pathways mediated by ERK, JNK, and p38 protein kinases. *Science*.

[41] Zhang L., Huang Y., Wu C. (2019). Network pharmacology based research on the combination mechanism between escin and low dose glucocorticoids in anti-rheumatoid arthritis. *Frontiers in Pharmacology*.

[42] Laragione T., Harris C., Gulko P. S. (2019). TRPV2 suppresses Rac1 and RhoA activation and invasion in rheumatoid arthritis fibroblast-like synoviocytes. *International Immunopharmacology*.

[43] Garcia S., Conde C. (2015). The role of poly(ADP-ribose) polymerase-1 in rheumatoid arthritis. *Mediators of Inflammation*.

[44] Okamoto K., Kobayashi T., Kobata T. (2000). Fas-associated death domain protein is a Fas-mediated apoptosis modulator in synoviocytes. *Rheumatology*.

[45] Itoh K., Hase H., Kojima H., Saotome K., Nishioka K., Kobata T. (2004). Central role of mitochondria and p53 in Fas-mediated apoptosis of rheumatoid synovial fibroblasts. *Rheumatology*.

[46] Lee J., Jeong H., Park E.-J. (2013). CIP2A facilitates apoptotic resistance of fibroblast-like synoviocytes in rheumatoid arthritis independent of c-Myc expression. *Rheumatology International*.

[47] Holmgren A., Lu J. (2010). Thioredoxin and thioredoxin reductase: current research with special reference to human disease. *Biochemical and Biophysical Research Communications*.

[48] Li Y., He N., Shen L., Liu M. (2019). Apoptotic effect of Aralia echinocaulis extract on fibroblast-like synoviocytes in rats with adjuvant-induced arthritis via inhibiting the Akt/Hif-1*α* signaling pathway in vitro. *Journal of Pharmacological Sciences*.

[49] Jiao Y., Ding H., Huang S. (2018). Bcl-XL and Mcl-1 upregulation by calreticulin promotes apoptosis resistance of fibroblast-like synoviocytes via activation of PI3K/Akt and STAT3 pathways in rheumatoid arthritis. *Clinical and Experimental Rheumatology*.

[50] Sun J., Yan P., Chen Y. (2015). MicroRNA-26b inhibits cell proliferation and cytokine secretion in human RASF cells via the Wnt/GSK-3beta/beta-catenin pathway. *Diagnostic Pathology*.

[51] Huang X.-Y., Zhang X.-M., Chen F.-H. (2014). Anti-proliferative effect of recombinant human endostatin on synovial fibroblasts in rats with adjuvant arthritis. *European Journal of Pharmacology*.

[52] Zhou J., Kvetnansky R., Radikova Z. (2005). Hormone concentrations in synovial fluid of patients with rheumatoid arthritis. *Clinical and Experimental Rheumatology*.

[53] Dziedziejko V., Kurzawski M., Safranow K., Chlubek D., Pawlik A. (2011). The effect of ESR1andESR2gene polymorphisms on the outcome of rheumatoid arthritis treatment with leflunomide. *Pharmacogenomics*.

[54] Gubbels Bupp M. R., Jorgensen T. N. (2018). Androgen-induced immunosuppression. *Frontiers in Immunology*.

[55] Karlson E. W., Chibnik L. B., McGrath M. (2009). A prospective study of androgen levels, hormone-related genes and risk of rheumatoid arthritis. *Arthritis Research & Therapy*.

[56] Zhang Y., Qiu H., Zhang H., Wang L., Zhuang C., Liu R. (2013). Vascular endothelial growth factor A (VEGFA) polymorphisms in Chinese patients with rheumatoid arthritis. *Scandinavian Journal of Rheumatology*.

[57] Jimi E., Fei H., Nakatomi C. (2019). NF-kappaB signaling regulates physiological and pathological chondrogenesis. *International Journal of Molecular Sciences*.

[58] Haleagrahara N., Miranda-Hernandez S., Alim M. A., Hayes L., Bird G., Ketheesan N. (2017). Inhibitory effect of quercetin in collagen-induced arthritis. *Biomedicine & Pharmacotherapy*.

[59] Kawaguchi K., Kaneko M., Miyake R., Takimoto H., Kumazawa Y. (2019). Potent inhibitory effects of quercetin on inflammatory responses of collagen-induced arthritis in mice. *Endocrine, Metabolic & Immune Disorders-Drug Targets*.

[60] Lee C. J., Moon S. J., Jeong J. H. (2018). Kaempferol targeting on the fibroblast growth factor receptor 3-ribosomal S6 kinase 2 signaling axis prevents the development of rheumatoid arthritis. *Cell Death Diseases*.

[61] Chen S., Yang Y., Feng H., Wang H., Zhao R., Liu H. (2014). Baicalein inhibits interleukin-1*β*-induced proliferation of human rheumatoid arthritis fibroblast-like synoviocytes. *Inflammation*.

[62] Liu R., Hao D., Xu W. (2019). *β*-Sitosterol modulates macrophage polarization and attenuates rheumatoid inflammation in mice. *Pharmaceutical Biology*.

[63] Doss H. M., Samarpita S., Ganesan R., Rasool M. (2018). Ferulic acid, a dietary polyphenol suppresses osteoclast differentiation and bone erosion via the inhibition of RANKL dependent NF-*κ*B signalling pathway. *Life Sciences*.

[64] Dinesh P., Rasool M. (2019). Berberine mitigates IL-21/IL-21R mediated autophagic influx in fibroblast-like synoviocytes and regulates Th17/Treg imbalance in rheumatoid arthritis. *Apoptosis*.

[65] Nishimoto N., Kishimoto T. (2006). Interleukin 6: from bench to bedside. *Nature Clinical Practice Rheumatology*.

[66] Elandt K., Aletaha D. (2011). Treating rheumatic patients with a malignancy. *Arthritis Research & Therapy*.

[67] Lee J. Y., Kim G. J., Choi J. K. (2018). 4-(Hydroxymethyl)catechol extracted from fungi in marine sponges attenuates rheumatoid arthritis by inhibiting PI3K/akt/NF-kappaB signaling. *Frontiers in Pharmacology*.

[68] Song B., Li X.-F., Yao Y. (2019). BMP9 inhibits the proliferation and migration of fibroblast-like synoviocytes in rheumatoid arthritis via the PI3K/AKT signaling pathway. *International Immunopharmacology*.

[69] Xu F.-B., Qiu H.-Y. (2018). Effects of artesunate on chondrocyte proliferation, apoptosis and autophagy through the PI3K/AKT/mTOR signaling pathway in rat models with rheumatoid arthritis. *Biomedicine & Pharmacotherapy*.

[70] Wallach D. (2016). The cybernetics of TNF: old views and newer ones. *Seminars in Cell & Developmental Biology*.

[71] van den Berg W. B., Miossec P. (2009). IL-17 as a future therapeutic target for rheumatoid arthritis. *Nature Reviews Rheumatology*.

[72] Pan F., Zhu L., Lv H., Pei C. (2016). Quercetin promotes the apoptosis of fibroblast-like synoviocytes in rheumatoid arthritis by upregulating lncRNA malat1. *International Journal of Molecular Medicine*.

[73] Guazelli C. F. S., Staurengo-Ferrari L., Zarpelon A. C. (2018). Quercetin attenuates zymosan-induced arthritis in mice. *Biomedicine & Pharmacotherapy*.

[74] Kim H.-R., Kim B.-M., Won J.-Y. (2019). Quercetin, a plant polyphenol, has potential for the prevention of bone destruction in rheumatoid arthritis. *Journal of Medicinal Food*.

[75] Lee D., Li N., Liu Y. (2018). Kaempferol inhibits the migration and invasion of rheumatoid arthritis fibroblast-like synoviocytes by blocking activation of the MAPK pathway. *International Immunopharmacology*.

[76] Yoon H.-Y., Lee E.-G., Lee H. (2013). Kaempferol inhibits IL-1*β*-induced proliferation of rheumatoid arthritis synovial fibroblasts and the production of COX-2, PGE2 and MMPs. *International Journal of Molecular Medicine*.

